# Interfacial Engineering of BiVO_4_ Immobilized on Sodium Alginate Aerogels Enable Synergistic Photocatalytic-Peroxymonosulfate Degradation of Rhodamine B

**DOI:** 10.3390/polym17162204

**Published:** 2025-08-12

**Authors:** Weidi Zhang, Tiantian Zhou, Jianhao Qiu

**Affiliations:** Jiangsu Co-Innovation Center of Efficient Processing and Utilization of Forest Resources, College of Chemical Engineering, Nanjing Forestry University, Nanjing 210037, China

**Keywords:** BiVO_4_, photocatalysis, dye degradation, sodium alginate, aerogel

## Abstract

The practical application of powdered photocatalysts is significantly hindered by challenges in recyclability and structural instability. This work proposes a sustainable immobilization strategy by integrating BiVO_4_ nanoparticles into a sodium alginate (SA) aerogel scaffold through a facile freeze-drying approach. The abundant hydroxyl/carboxyl groups of SA enable uniform dispersion of BiVO_4_ within the porous network, while the aerogel architecture enhances light-harvesting efficiency and mass transfer kinetics. Innovatively, peroxymonosulfate (PMS) was introduced to synergistically couple photocatalysis with sulfate radical-based advanced oxidation processes (SR-AOPs), where the photogenerated electrons from BiVO_4_ effectively activate PMS to yield high-activity ·SO_4_^−^ radicals. The optimized BiVO_4_/SA aerogel achieves nearly complete removal of Rhodamine B within 2 h under visible light, which is competitive to pure BiVO_4_ powders. In addition, the mechanically robust aerogel exhibits exceptional reusability, retaining ~90% efficiency after five cycles without structural collapse. This work provides a paradigm for designing recyclable photocatalyst carriers with dual oxidation pathways, demonstrating significant potential for industrial wastewater treatment.

## 1. Introduction

Dyes, which are typical contaminants in wastewater, have been incontrovertibly established as a peril to the sustainability of life forms [1,2,3]. Amidst the escalating crisis of environmental pollution, numerous strategies for pollutant removal have been developed [4,5,6]. Among them, advanced oxidation processes (AOPs), encompassing photocatalysis, electrochemistry, and Fenton/Fenton-like reactions, are regarded as promising avenues for the complete degradation and mineralization of organic pollutants [7,8,9]. Photocatalysis has garnered significant attention owing to its harnessing of sunlight, mild operational conditions, and alignment with green development principles [10,11]. Recently, sulfate radical-based AOPs (SR-AOPs), leveraging sulfate radicals (·SO_4_^−^), have captured widespread interest [12,13]. The formidable redox potential of ·SO_4_^−^ (ranging from 2.6 to 3.1 eV) underscores its potent oxidative capacity [7,14]. While ·SO_4_^−^ can be generated through the activation of peroxymonosulfate (PMS), conventional activation methods, such as heat, ultrasound, UV radiation, gamma irradiation, and high pH conditions, are often hindered by drawbacks like high costs, propensity for side reactions, and sludge generation [14,15,16]. Photocatalytic activation of PMS presents a viable solution, transcending these limitations. The integration of photocatalytic technology with SR-AOPs for wastewater pollutant remediation has thus emerged as a research frontier [13,17,18].

As one of the typical photocatalysts, BiVO_4_ has the characteristics of a narrow band gap and superb visible light response [19,20,21]. To enhance its photocatalytic ability, various strategies have been proposed, including the fabrication of heterostructures, elemental doping, and precise morphology manipulation [22,23,24]. For example, Yi et al. harnessed an innovative microemulsion-calcination approach to achieve in situ growth of BiOCl nanosheets onto BiVO_4_ surfaces, thereby constructing a 0D/2D p-n heterojunction. This design effectively mitigated the recombination of photogenerated charge carriers, obtaining a remarkable achievement of 99.5% efficient degradation of Rhodamine B (RhB) [25]. Despite their promising properties, photocatalysts in powdered form often suffer from poor recyclability and reuse inconvenience in practical applications, compounded by a tendency towards aggregation [26,27]. To mitigate these drawbacks, immobilizing BiVO_4_ onto a suitable support/host emerges as an effective strategy [28,29]. Sodium alginate (SA), a natural polysaccharide comprising *β*-D-mannuronic acid and *α*-L-guluronic acid, boasts biocompatibility, hydrophilicity, biodegradability, non-toxicity, and abundant availability [30]. The unique properties of the good degradability and the presence of numerous oxygen-containing groups (-OH and -COOH) endow it with a high affinity towards catalyst particles [31,32]. Previously, Akash et al. demonstrated the fabrication of kaolinite/g-C_3_N_4_-alginate beads, which exhibited remarkable performance in removing 97% of brilliant green (10 mg/L) within 90 min under visible light irradiation. This enhanced performance was attributed to improved adsorption capabilities and efficient utilization of visible light. Furthermore, the beads maintained a dye removal efficiency of 82% even after ten cycles, underscoring their excellent recyclability [33]. Similarly, Liu et al. successfully immobilized ZnIn_2_S_4_ onto SA to create a foam catalyst, achieving a photocatalytic reduction efficiency of 93% for Cr(VI). This composite material not only retained its superior photocatalytic performance but also maintained a relatively intact structure after six reuse cycles, highlighting its potential for sustainable applications [28].

**Figure 1:**
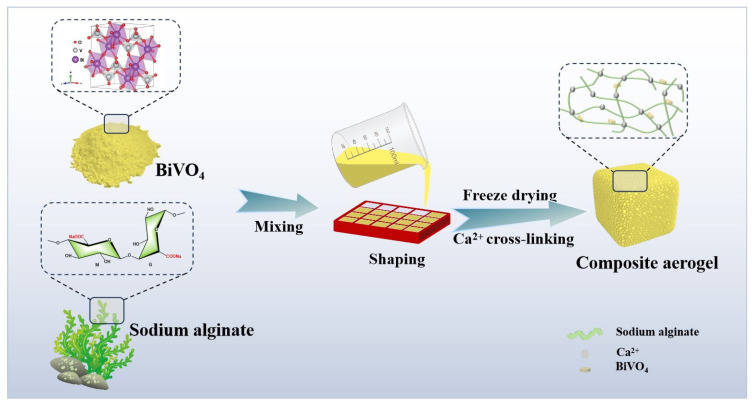
Schematic diagram for the fabrication of BiVO_4_@SA aerogels.

Herein, BiVO_4_/SA composite aerogels with a porous structure and uniform BiVO_4_ distribution were fabricated (Figure 1). The obtained composites were combined with PMS technology for the photocatalytic degradation of RhB. In the RhB photocatalytic removal experiment, the RhB removal rate reached 99.4% after 2 h of illumination. In addition, the sample has good recyclability and reusability, with it still maintaining a high removal rate of RhB (87.8%) after five cycles.

## 2. Materials and Methods

### 2.1. Reagents

Ammonium metavanadate (NH_4_VO_3_, 99%) was supplied by Shanghai Macklin Biochemical Co., Ltd. (China). Bismuth trichloride (BiCl_3_, AR) and ethanolamine (H_2_NCH_2_CH_2_OH, 99%) were purchased from Aladdin Industrial Company (China). Sodium alginate (SA, CP), anhydrous calcium chloride (CaCl_2_), and methanol (99.5%) were obtained from Sinopharm Chemical Reagent Co., Ltd. (China). Rhodamine B (RhB) was procured from Tianjin Chemical Reagent Co., Ltd. (China).

### 2.2. Synthesis of Photocatalysts

BiVO_4_ was obtained according to the preparation steps reported in the literature, with some modifications [34]. First, 1 mmol BiCl_3_ was dispersed in 100 mL of deionized water (DI) and stirred to form a suspension. Subsequently, 1 mmol NH_4_VO_3_ was added to the above suspension. After that, 0.5 mL of 1 M ethanolamine aqueous solution was added to adjust the pH to neutral. Thereafter, the mixture was transferred to a polytetrafluoroethylene-lined autoclave and reacted at 160 °C for 12 h. The yellow powder was obtained by means of centrifugation, washing with deionized water and ethanol six times, and drying at 60 °C in a vacuum oven overnight.

For the fabrication of BiVO_4_/SA composite aerogels, first, the pre-synthesized BiVO_4_ was dispersed in 20 mL of deionized water and ultrasonically treated for 20 min. Subsequently, sodium alginate powder (0.4 g) was slowly added to the above suspension under magnetic stirring. After stirring for 4 h, the resulting homogeneous solution was evenly poured into a silica mold with the help of an oscillator and freeze-drying at −65 °C overnight. Next, the obtained foam was soaked in 3 wt% CaCl_2_ for cross-linking, washed with deionized water to remove excess Ca^2+^, and freeze-dried again to prepare composite aerogels. Based on the mass ratio of BiVO_4_ to sodium alginate (0.25, 0.5, 1, and 2), the composite aerogels were named BSA-x (x = 1, 2, 3, and 4). Pure sodium alginate aerogels were synthesized following the same procedure, with the exception of the addition of BiVO_4_, and were labeled as SA.

### 2.3. Characterizations

An X-ray diffractometer (Ultima IV, Japan) was applied to determine the X-ray diffraction (XRD) patterns. The scanning electron microscopy (SEM) and energy-dispersive X-ray spectroscopy (EDS) mapping images were tested through JSM-7600F (JEOL, Japan). A Fourier transform infrared (FT-IR) spectrometer (VERTEX 80 V, USA) recorded the FT-IR spectra. X-ray photoelectron spectroscopy (XPS) was performed via an electron spectrometer (AXIS UltraDLD, Japan). To obtain the UV-vis diffuse reflectance spectroscopy (DRS), the Shimadzu UV-2600 instrument (Japan) was employed. On a CHI 760E electrochemical workstation using a standard three-electrode system, the electrochemical properties of the obtained composites involving transient photocurrent response and electrochemical impedance spectroscopy (EIS) analyses were carried out. N_2_ sorption curves were obtained using a TriStar II (Micromeritics, USA) instrument, and the specific surface area was calculated through the Brunauer–Emmett–Teller (BET) method. Steady-state photoluminescence (PL) and time-resolved PL decay spectra were determined through a FluoroMax-4 instrument. Zeta potentials were measured using the ZETASIZER Nano-ZS from Malvern Instruments (UK).

### 2.4. Photocatalytic Degradation Tests

The photocatalytic activity of the obtained composite aerogels was evaluated by degrading the RhB under the irradiation of a xenon lamp (CEL-HXF300, China, light intensity: 100 mW·cm^−2^). For each experimental procedure, 40 mL of dye solution, 40 mg of photocatalyst, and a certain amount of PMS were added to the reactor, and the whole system was kept under stirring and condensed water circulation to maintain the room temperature. In order to achieve adsorption–desorption equilibrium, the reaction system was stirred in the dark for 1 h before irradiation. To determine the RhB concentration, 2.5 mL of the reaction liquid was taken out with a syringe every 30 min, and the catalyst was filtered out with a 0.22 μm filter to obtain the filtrate. The obtained liquid was transferred to a quartz cuvette, and the absorbance at the corresponding maximum absorption wavelength (554 nm for RhB) was measured on a UV-vis spectrophotometer (UV-2600, Japan). Finally, the RhB concentration was obtained by means of a specific standard curve. Under the same experimental conditions, carbon tetrachloride (CCl_4_), disodium edetate (EDTA-2Na), benzoquinone (BQ), isopropyl alcohol (IPA) and tert-butanol (TPA) were introduced as scavengers for electrons (e^−^), holes (h^+^), superoxide radicals (·O_2_^−^), hydroxyl radicals (·OH) and ·SO_4_^−^ to explore the photocatalytic mechanism of the photocatalyst.

## 3. Results and Discussion

### 3.1. Aerogel Characterizations

**Figure 2:**
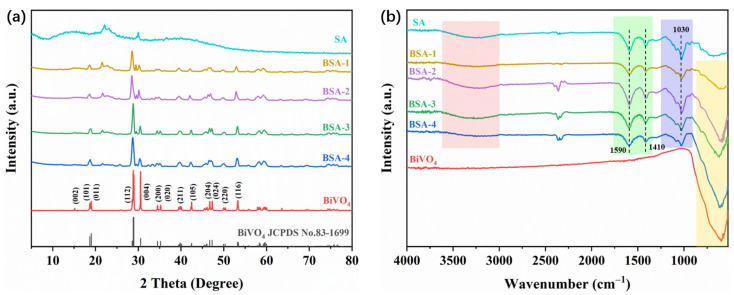
XRD patterns (**a**) and FT-IR spectra (**b**) of the prepared composites.

The crystalline phase of the synthesized BiVO_4_ and BiVO_4_/SA composite aerogel was analyzed by means of X-ray diffraction. As shown in Figure 2a, it can be clearly seen that BiVO_4_ has good crystallinity, among which the characteristic peaks at 18.7°, 19.1°, 29.0°, 30.6°, 34.6°, 35.2°, 39.7°, and 42.4° correspond to the (101), (011), (112), (004), (200), (020), (211), and (105) crystal planes [34]. The characteristic peaks are highly similar to those of the monoclinic phase of BiVO_4_ (JCPDS: 83-1699), indicating that the monoclinic phase of BiVO_4_ has been successfully synthesized. The estimated crystallite size of BiVO_4_ was ca. 210 nm through the Scherrer equation. In the spectrum of pure SA, the broad peaks at 14.1°, 22.2°, 23.3°, and 29.9° show its low crystallinity [28]. In the composite BSA-x aerogel, the characteristic peaks of monoclinic BiVO_4_ are well preserved, while the characteristic peaks of SA can also be seen intuitively, and the intensity of the characteristic peaks decreases with the increase in BiVO_4_ content. In addition, the characteristic peaks of BiVO_4_ have a certain offset in the composite aerogel, such as the characteristic peaks at 18.7° and 19.1° shifting to the left and forming a small broad peak, which may be the lattice distortion caused by the introduction of SA, and this also proves the successful synthesis of the composite aerogel [22].

In order to further determine the chemical structure and composition of the BiVO_4_/SA composite aerogel, an FT-IR test was performed, and the results are shown in Figure 2b. The broad absorption band of pure SA aerogel near 3000–3600 cm^−1^ originates from the hydroxyl group of the alginate skeleton. The bands at 1410 cm^−1^ and 1590 cm^−1^ correspond to the asymmetric and symmetric stretching vibrations of the carboxyl groups present in sodium alginate, indicative of the material’s composition [28]. The absorption band at 1030 cm^−1^ is the C-O-C of the glycosidic bond in the SA chain [35]. For the BiVO_4_/SA composite aerogel, these characteristic peaks belonging to SA are well retained and have slight shifts, which may be the strong interaction between the hydroxyl groups of SA and BiVO_4_. The absorption bands at 600 cm^−1^ and 730 cm^−1^ belong to the V-O symmetric and asymmetric stretching vibrations of BiVO_4_ [36]. Absorption bands of V-O symmetric and asymmetric stretching vibrations can be observed in the composite aerogels, and the intensity of the absorption bands increases with the increase in BiVO_4_ content. Similarly, the position of the absorption band belonging to BiVO_4_ also experiences a slight shift. The above analysis proves that there is a strong interaction between the catalyst and the matrix, which will be conducive to the effective separation of photogenerated electron–hole pairs in the composite aerogel.

**Figure 3:**
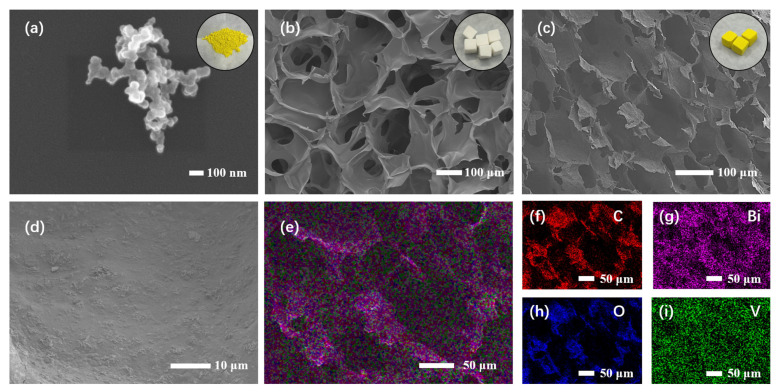
SEM images of BiVO_4_ (**a**), SA (**b**), and BSA-4 (**c**,**d**) and EDS mapping images of BSA-4 (**e**–**i**).

In order to analyze the microstructure of the composite aerogel, SEM tests were conducted, and the corresponding results are shown in Figure 3a–d. It can be observed that pure BiVO_4_ is in the form of nanoparticles with an average size of about 200 nm, which is consistent with the results reported in the literature [22,34]. In Figure 3b, the cross-section of the blank SA sample shows a smooth surface and rich macroporous structure. In the cross-sectional view of the composite aerogel (Figure 3c), BSA-4 inherits the rich macroporous structure of SA, but shows a rough surface, which is most likely because BiVO_4_ enters the matrix of SA and wraps around the SA chain. In addition, in Figure 3d, BiVO_4_ is evenly distributed on SA. At a larger magnification, the morphology of BiVO_4_ is retained in the composite aerogel BSA-4. Therefore, the composite aerogel not only obtains a highly porous matrix but also combines nanoparticle photocatalysts, which can expose more active sites to improve removal efficiency [37]. Furthermore, the integration of the powdered photocatalyst within the SA matrix to create an aerogel significantly enhances the catalyst’s facile utilization and recyclability for practical applications. Additionally, the energy-dispersive X-ray spectroscopy (EDS) analysis of BSA-4, as shown in Figure 3e–i, confirms the uniform distribution of elements Bi, V, O, and C across the composite, thereby substantiating the successful synthesis of the BiVO_4_/SA composite aerogel.

**Figure 4:**
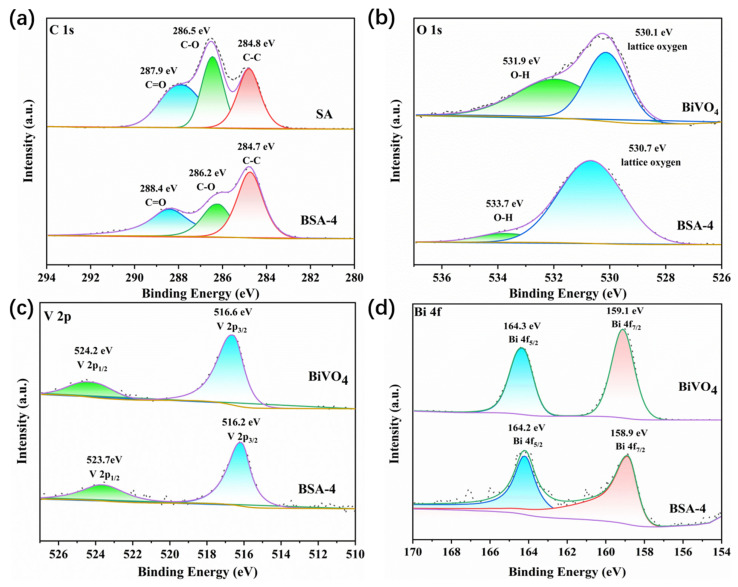
XPS spectra of different samples: C 1s (**a**), O 1s (**b**), V 2p (**c**), and Bi 4f (**d**).

The chemical element composition and valence state of BiVO_4_, SA, and BiVO_4_/SA composite aerogels were studied by means of XPS. As shown in Figure 4a, the C 1s spectrum of SA can be fitted into three peaks at 284.8 eV, 286.5 eV, and 287.9 eV, corresponding to C-C, C-O, and C=O in sodium alginate, respectively [38]. For the composite aerogel BSA-4, the C 1s spectrum is similar to that of SA, and the peak positions of C-C, C-O, and C=O are slightly shifted to 284.7 eV, 286.2 eV, and 288.4 eV, respectively. In the O 1s spectrum, the peak at 530.1 eV (Figure 4b) belongs to the lattice oxygen of the layered Bi_2_O_2_^2+^ of BiVO_4_, while the characteristic peak at 531.9 eV is derived from the O-H group of water adsorbed on the sample surface [39]. For the composite aerogel BSA-4, the characteristic peak position of the lattice oxygen of the layered Bi_2_O_2_^2+^ shifted to 530.7 eV. The increase in binding energy demonstrated the decrease in electron density, which can be attributed to the interaction between SA and BiVO_4_. In the high-resolution O 1s spectrum of BSA-4, the characteristic peak at 533.7 eV is the O-H group of water adsorbed on the surface of the material. For the V 2p spectrum (Figure 4c), the characteristic peak position corresponding to the V 2p_3/2_ and V 2p_1/2_ orbitals of V^5+^ in the composite aerogel shifted from 516.6 eV and 524.2 eV in BiVO_4_ to 516.2 eV and 523.7 eV, respectively [40]. The decrease in binding energy shows the increase in electron density. In addition, in the Bi 4f spectrum of BiVO_4_, the peaks at 164.71 eV and 159.44 eV belong to Bi 4f_5/2_ and Bi 4f_7/2_, respectively, which proves the existence of Bi^3+^ (Figure 4d) [41]. In the high-resolution Bi 4f spectrum of BSA-4, the peaks assigned to Bi 4f_5/2_ and Bi 4f_7/2_ moved to 164.2 eV and 158.9 eV, respectively, which shows that BiVO_4_ and SA are tightly bound. In summary, there is a strong interaction between BiVO_4_ and SA in BSA-4, which causes the peak shifts of each element in the XPS spectra. This further confirms the high interface bonding strength between BiVO_4_ and SA, and the tightly bound interface is conducive to the separation and migration of photogenerated carriers, which can improve photocatalytic activity [28].

Thermogravimetric analysis (TGA) under a nitrogen atmosphere was employed to study the thermal stability of the aerogels. The weight loss below 100 °C in the aerogels was primarily due to the loss of adsorbed water (Supporting Information, Figure S1). The significant weight decline that appeared from 200 to 550 °C was caused by the hydrogen bond fracture as well as the decomposition of the SA skeletal structure. Furthermore, the composite aerogel BSA-4 inherited the excellent thermal stability characteristic of BiVO_4_, as evidenced by its high residual mass after thermal treatment. Quantitative analysis of the residual weight at 800 °C, presumably through thermogravimetric analysis (TGA), revealed that the content of BiVO_4_ in the hybrid aerogel BSA-4 is 65.4%, a value that closely aligns with the theoretical composition.

It is well known that a high-performance aerogel-based photocatalyst should exhibit superior mechanical properties to support the regeneration process. In this study, the compressibility of aerogels was investigated through compressive experiments. The compressive strength of composite aerogels increased with the increase in BiVO_4_ content (Supporting Information, Figure S2). The compression deformation process of BSA-x aerogels can be divided into two stages: (1) the dense micropores within BSA-x provide sufficient space for compression, and (2) the nanoparticles firmly anchored on the pore walls enhance the stability of the aerogels’ skeletal structure, effectively dispersing stress and aiding in structural recovery after compression [42]. Thus, the incorporation of BiVO_4_ nanoparticles not only prevents the aerogel from severe structural collapse but also contributes to the rapid recovery of the pore structure after compression.

**Figure 5:**
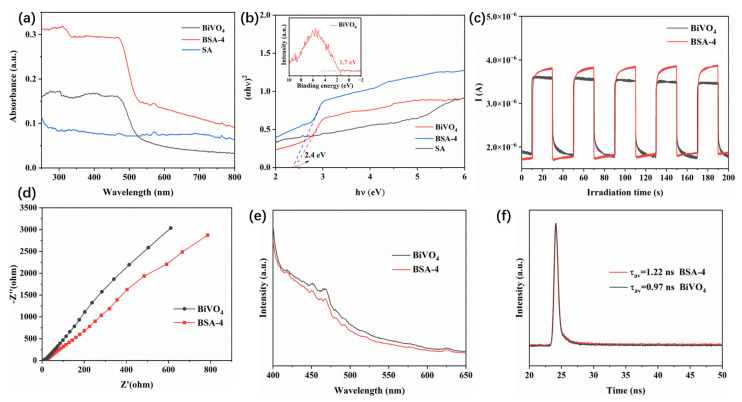
UV-vis diffuse reflectance spectra of different samples (**a**), (αhν)^2^ vs. hν curves and XPS valence band spectrum (**b**), photocurrent spectra (**c**), EIS Nyquist plots (**d**), PL (**e**), and time-resolved PL decay (**f**) spectra.

The performance and removal efficiency of photocatalysts are intrinsically linked to the structure of their band gap. In this study, the light absorption properties and band gap structure of the synthesized composite materials were investigated using UV-vis absorption spectra. As shown in Figure 5a, BiVO_4_ exhibits strong absorption in the UV-visible region, with an absorption edge of approximately 550 nm. The composite aerogel BSA-4 had similar light response ability and absorption edge, while exhibiting stronger absorption intensity, which suggested that the composite aerogel BSA-4 has enhanced light absorption ability. This can be attributed to the layered porous structure formed in the aerogel, which is conducive to scattering the absorbed light, thereby improving the utilization rate of light. The band gap of BiVO_4_ and BSA-4 is calculated by converting the diffuse reflectance results using the Tauc diagram method: (*α*h*ν*)^n^ = A(h*ν* − *E_g_*) [43]. As shown in Figure 5b, the *E_g_* value of BiVO_4_ and BSA-4 are calculated to be 2.40 eV and 2.37 eV, respectively. From the XPS spectrum in Figure 5b, the E_VB-XPS_ potential of BiVO_4_ is 1.7 eV. By combining the following empirical formulas, *E_VB_* = *E_VB-XPS_* + φ − 4.44 (the work function of the instrument φ = 4.43 eV) and *E_CB_* = *E_VB_* − *E_g_*, the VB and CB potentials of BiVO_4_ were calculated to be 1.69 eV and −0.71 eV, respectively. The separation ability and transfer efficiency of photogenerated electron–hole pairs are important factors affecting photocatalytic performance. To this end, transient photocurrent response tests and electrochemical impedance spectroscopy (EIS) tests were used to evaluate the photocatalytic performance of the composite aerogels. As shown in Figure 5c, the composite aerogel BSA-4 exhibited a stronger photocurrent response, indicating that it has a higher separation efficiency of photogenerated electron–hole pairs. Moreover, BSA-4 also exhibited a smaller Nyquist plot arc radius (Figure 5d), which proves that charge transfer is easier in the composite aerogel. Additionally, the lower PL intensity (Figure 5e) and higher fluorescence lifetime (Figure 5f) of BSA-4 further confirm its efficient inhibition of charge carrier recombination.

### 3.2. Photocatalytic Tests

**Figure 6:**
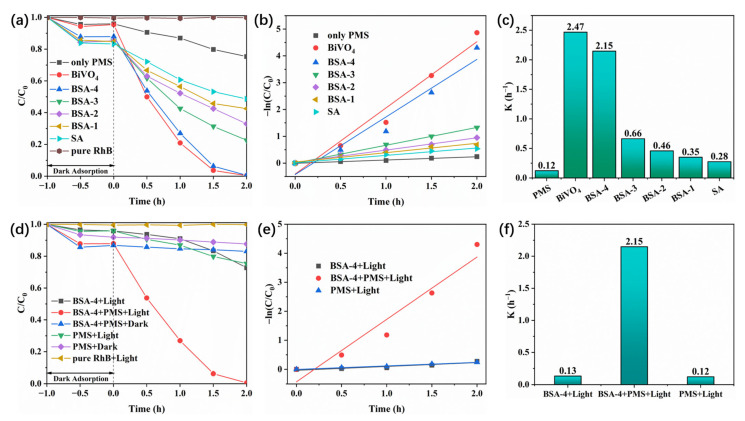
Photocatalytic degradation efficiency of different samples of RhB: corresponding kinetic curves and constants (**a**–**c**) and the effects of PMS, light, and composite aerogels on the photocatalytic removal efficiency of RhB and the corresponding kinetic curves and constants (**d**–**f**). The initial reaction pH value is 6.

The photocatalytic activation of PMS over the BiVO_4_/SA composite aerogel for dye removal was evaluated under simulated sunlight. The entire reaction mixture was kept in the dark for 60 min to establish adsorption–desorption equilibrium. Initially, the photocatalytic degradation performance of BiVO_4_, SA, and BSA-x was evaluated in the presence of PMS. As shown in Figure 6a, RhB exhibits negligible self-degradation in the absence of the composite aerogel or PMS, indicating that the dye’s self-degradation can be disregarded. In a system containing only PMS, the RhB removal rate was a mere 24.7%. Upon the addition of BiVO_4_, SA, and BSA-x, there was a significant enhancement in the RhB removal rate. Moreover, the RhB removal rate is observed to increase progressively as the amount of BiVO_4_ added increases. Among all of the samples, the removal rate of RhB by the best sample, BSA-4, reached 99.4% after 2 h of light illumination, which is 1.93 times the removal rate of RhB by SA. At the same time, the removal rate of RhB by BiVO_4_ after two hours of illumination was 99.6%, which was similar to that of BSA-4. This means that BSA-4 is a better catalyst because it contains less BiVO_4_. However, aerogel as a carrier can greatly enhance the interface contact of the supported photocatalyst and significantly reduce the agglomeration of photocatalyst particles, which provides more surface active sites for capturing pollutants. Moreover, the structure of the aerogel enhances the light scattering ability and reduces the diffusion distance of electrons, thereby improving the photocatalytic activity. Meanwhile, the apparent reaction rate constant of the sample to pollutants was calculated using the pseudo-first-order kinetic model (−ln (C/C_0_) = kt; this model is workable here since a low RhB concentration (≤50 mg/L) was adopted and the catalyst concentration and light intensity were constant during photocatalysis), and the results are shown in Figure 6b,c. It can be seen that the kinetic constant of BSA-4 is 2.15 h^−1^, which is the highest among all BSA-x samples and is close to the kinetic constant of BiVO_4_ (2.47 h^−1^).

Based on previous experiments, we evaluated the effects of PMS, light, and the composite aerogel on the photocatalytic effect in this system. As shown in Figure 6d, in the absence of PMS, the degradation rate of RhB over BSA-4 was only 27.3%, because the concentration of RhB was too high and the photocatalytic activity of the composite aerogel was limited. After adding PMS, the removal rate increased to 99.4%. In the case of only PMS, the removal rate of RhB was only 24.7%, which can be attributed to the slow reaction rate of unactivated PMS. Moreover, the effect of light in the system was evaluated. PMS could not be fully activated under light, so the degradation effect on RhB was not much different from that of PMS in the dark. Furthermore, in the BSA-4+PMS system, without light, photogenerated electrons and holes in BSA-4 could not be generated, and PMS could not be activated to produce ·SO_4_^−^ free radicals to participate in the RhB degradation reaction. Similarly, we calculated the kinetic constants of the samples for pollutants using the pseudo-first-order kinetic model (−ln(C/C_0_) = kt) (Figure 6e,f). Under illumination, the kinetic constant of BSA+PMS is 2.15 h^−1^, which is 16.5 times that of BSA-4 (0.13 h^−1^) and 17.9 times that of PMS (0.12 h^−1^). In summary, we experimentally determined that the removal effect of RhB can be greatly improved when PMS, illumination, and the composite aerogel are present in the photocatalytic system.

**Figure 7:**
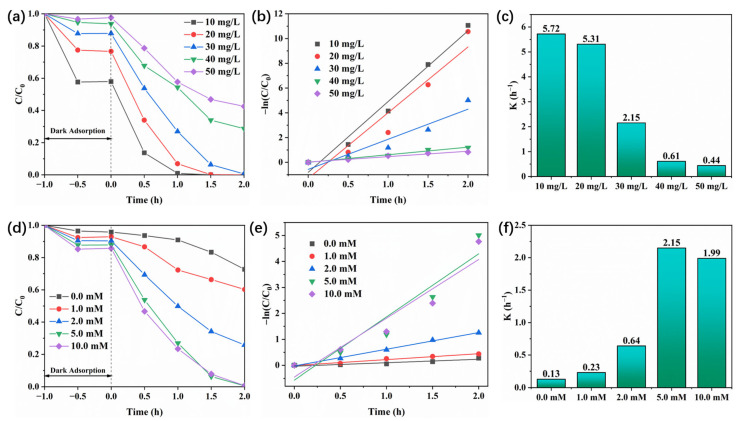
Effect of RhB concentration on the photocatalytic performance of BSA-4 and the corresponding kinetic curves and rate constants (**a**–**c**), and effect of PMS concentration on the photocatalytic performance of BSA-4 and the corresponding kinetic curves and rate constants (**d**–**f**). The initial reaction pH value is 6.

Besides, the effect of dye concentration on the photocatalytic removal performance was investigated. As shown in Figure 7a–c, as the initial concentration of RhB increases, the removal rate gradually decreases. The kinetic constant also decreases with increasing concentration. On the one hand, this is because the number of available active sites and photogenerated electron–hole pairs is limited; on the other hand, the increase in RhB concentration makes the color of the reaction system solution darker, thereby reducing the light transmittance and inhibiting the generation of photogenerated electrons and holes. Meanwhile, the effect of PMS concentration on the removal effect of RhB was evaluated (Figure 7d). When the PMS concentration increases in the range of 0 to 5 mM (mmol/L), the degradation rate of RhB gradually increases, and the kinetic constant also increases from 0.13 to 2.15 h^−1^ (Figure 7e,f). When a 5 mM concentration of PMS was added, the removal rate of RhB reached 99.4%. When the PMS concentration was increased to 10 mM, the removal rate was 99.3%, and the kinetic constant decreased to 1.99 h^−1^. This is owing to the fact that although higher PMS concentrations produce more active radicals during the degradation process, the number of photogenerated electrons–holes produced by the photocatalyst is not enough to fully activate the PMS in the solution when 10 mM PMS is added [44,45].

**Figure 8:**
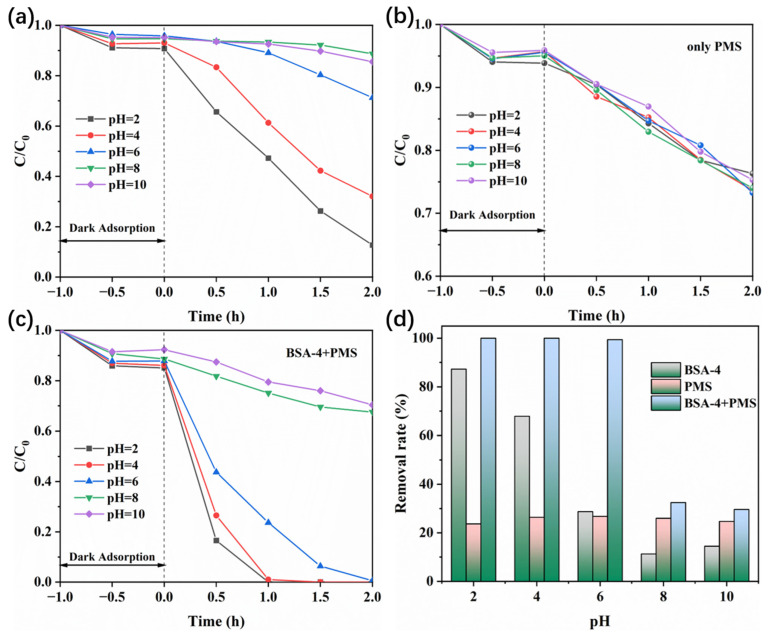
Effect of initial solution pH on RhB removal by BSA-4 (**a**), PMS (**b**), and BSA-4+PMS (**c**), and the corresponding total RhB removal rate (**d**).

Additionally, pH is an important parameter that affects the photocatalytic activity of the catalyst. It affects the surface properties of the material, the dissociation of dye molecules, and the formation of radicals [46]. The pH value of the initial RhB solution was adjusted by changing the amount of H_2_SO_4_ (SO_4_^2−^ would not interfere with PMS) and NaOH added. As shown in Figure 8a, without the addition of PMS, the degradation rate of RhB was greatly improved under acidic conditions, reaching 87.3% (pH = 2), which is consistent with previous literature reports [47]. This may be because the surface of the material is protonated and positively charged under acidic conditions, which is conducive to the transfer of photogenerated electrons to the surface of the material and combines with adsorbed oxygen to generate superoxide radicals (·O_2_^−^), thereby improving photocatalytic activity [48]. The zeta potentials of BiVO_4_ under different pH values were monitored (Figure S3), which proved that the photocatalyst is positively charged in an acidic environment. In the case of only PMS, when the initial pH changes in the range of 2–10, the removal effect of RhB does not change significantly, which is consistent with the results reported in the literature (Figure 8b) [49,50]. This may be because of the second acid dissociation constant of PMS, pKa = 9.4, so the addition of PMS acidifies the solution and fails to reach the alkaline conditions (pH > 11) [50]. In the BSA-4+PMS system, the combined effect of BSA-4 and PMS on the RhB removal rate was evaluated at various pH values. Under acidic conditions (pH = 2 and pH = 4), the removal rate of RhB achieved complete degradation of 100%, significantly outperforming the rates observed under alkaline conditions, which were 32.5% at pH = 8 and 29.6% at pH = 10 (Figure 8c). PMS exists in the solution in a negative form (HSO_5_^−^); thus, an acid environment is conducive to PMS activation due to the electrostatic interaction between PMS and photocatalysts. However, in an alkaline environment, plenty of OH^−^ would react with HSO_5_^−^, thereby decreasing the concentration of HSO_5_^−^ [49].

**Figure 9:**
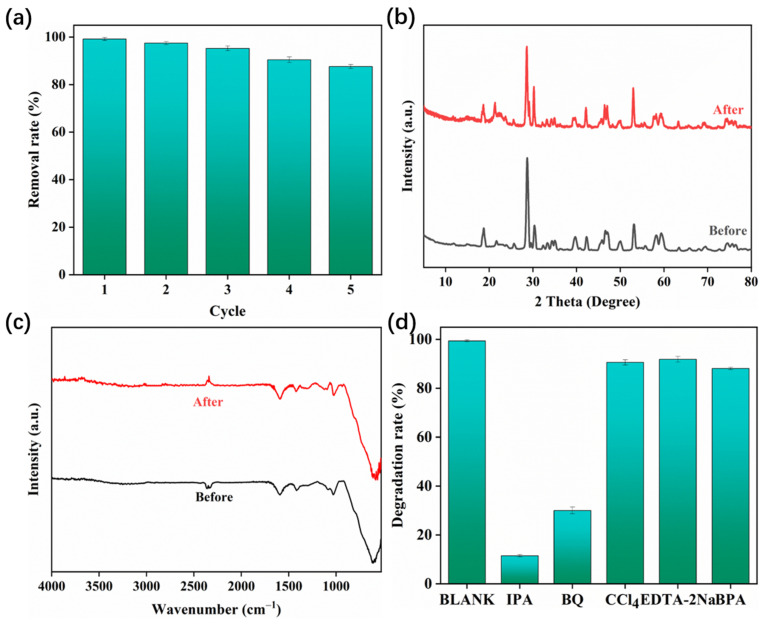
Cyclic experiments (**a**), XRD patterns (**b**), and FT-IR spectra (**c**) of the fresh and used BSA-4, reactive species quenching experiments for the photocatalytic elimination of RhB degradation (**d**).

In practical applications, the recyclability and reusability of the material are verified by means of cycling experiments. Compared with powder, the composite aerogel loaded with BiVO_4_ on SA can avoid the disadvantage of the complicated recovery process of powder catalysts. Specifically, after each cycle, the aerogel can be directly recovered from the aqueous solution and washed with deionized water and ethanol multiple times before reuse. As shown in Figure 9a, after five cycles, the photocatalytic removal efficiency of RhB remains above 87.8%. In addition, there is no obvious change in the XRD and FT-IR spectra of BSA-4 before and after the cycle, which indicates that the crystal structure and chemical structure of the composite aerogel are not destroyed (Figure 9b,c). In addition, the RhB degradation abilities of BSA-4 in the presence of various heavy metal ions were detected (Figure S4). As a result, under the interference of heavy metal ions, the degradation capacities have no obvious influence, demonstrating the high applicability of BSA-4. In order to explore the mechanism of the photocatalytic reaction, the active species of BSA-4 and PMS systems in the photocatalytic degradation reaction of RhB were determined by means of radical capture experiments, among which CCl_4_ (0.2 mM), EDTA-2Na (0.2 mM), IPA (0.2 mM), TPA (0.2 mM), and BQ (0.2 mM) were used as probe chemicals (Figure 9d). CCl_4_ and EDTA-2Na are electron and hole scavengers, respectively, and have a certain effect on the degradation process of RhB. BQ is a quencher of ·O_2_^−^; when it is added to the reaction system, the degradation of RhB is significantly inhibited, which shows that ·O_2_^−^ is the active species controlling the reaction. IPA is a capture agent of ·SO_4_^−^ and ·OH, while TBA can only effectively remove ·OH. As shown in Figure 9d, after the addition of IPA, the degradation rate of RhB decreased significantly, while the addition of TBA had almost no effect. This shows that ·SO_4_^−^ is also one of the main active species in the reaction.

In view of the energy band structure of BiVO_4_ and the free radical capture experiment, combined with the potentials of O_2_/O_2_^−^ (−0.33 eV) and H_2_O/·OH (+2.38 eV), we can propose the mechanism of the composite aerogel BSA-4 and PMS system in the degradation of RhB. The electrons (e^−^) on the VB of BiVO_4_ are excited to jump to the CB, leaving photogenerated holes (h^+^) on the VB and then the photogenerated electrons in BiVO_4_ are transferred to the catalyst surface and generate ·O_2_^−^ with oxygen. Because the VB potential of BiVO_4_ is 1.69 eV, the photogenerated holes in the VB cannot directly generate ·OH. Therefore, the photogenerated holes directly react with HSO_5_^−^ to generate ·SO_5_^−^, and the consumption of holes promotes the generation of electrons. The photogenerated electrons could react with HSO_5_^−^ to generate ·SO_4_^−^. Both ·O_2_^−^ and ·SO_4_^−^ would then degrade RhB, and finally mineralize it into H_2_O and CO_2_ [49]. The photocatalytic reaction process on BSA-4 is shown as follows [45,51]:

(1) $$BSA-4\overset{hv}{\to }h^{+}+e^{-}$$

(2) $$e^{-}+O_{2}\to {\cdot O}_{2}^{-}$$

(3) $${HSO}_{5}^{-}+e^{-}\to {\cdot SO}_{4}^{-}+{OH}^{-}$$

(4) $${HSO}_{5}^{-}+h^{+}\to {\cdot SO}_{5}^{-}+H^{+}$$

(5) $$2{\cdot SO}_{5}^{-}\to 2{\cdot SO}_{4}^{-}+O_{2}$$

## 4. Conclusions

In this study, a porous BiVO_4_/SA composite aerogel was fabricated through a freeze-drying technique. The polysaccharide scaffold of SA effectively functions as a mechanical stabilizer, facilitating the uniform distribution of BiVO_4_ within the matrix. To assess the photocatalytic ability of the composite aerogel, RhB was employed as a model contaminant. Notably, the BSA-4 sample exhibited superior photocatalytic activity, achieving a remarkable 99.4% degradation rate within 2 h, with the presence of PMS. Our results showed that ·O_2_^−^ and ·SO_4_^−^ are the main active species involved in the photocatalytic degradation of RhB. In addition, the shaped aerogel is easy to recycle, with good reusability. It maintains an 87.8% removal rate for RhB after five cycles, which means that the as-prepared aerogel is a promising wastewater purification material.

## Data Availability

The original contributions presented in this study are included in the article/Supplementary Material. Further inquiries can be directed to the corresponding author.

## Supplementary Materials

The following supporting information can be downloaded at https://www.mdpi.com/article/10.3390/polym17162204/s1. Figure S1. Thermogravimetric analysis (TGA) under a nitrogen atmosphere of different samples; Figure S2. Stress–strain curves of different samples; Figure S3. Zeta potentials of BiVO_4_ under different pH values; Figure S4. Photocatalytic degradation rates of BSA-4 for RhB in the presences of various heavy metal ions.
