# Supplementary material for: Interfacial Engineering of BiVO4 Immobilized on Sodium Alginate Aerogels Enable Synergistic Photocatalytic-Peroxymonosulfate Degradation of Rhodamine B
Source: Polymers (Basel). 2025 Aug 12;17(16):2204. doi: 10.3390/polym17162204 (PMC12389655; doi:10.3390/polym17162204)
Supplement: Supplementary file 1 [file polymers-17-02204-s001.zip › polymers-3762023-supplementary.pdf]

Supporting information

Interfacial Engineering of $BiVO_4$ Immobilized on Sodium Alginate Aerogels Enable Synergistic Photocatalytic-Peroxymonosulfate Degradation of Rhodamine B

Weidi Zhang, Tiantian Zhou, Jianhao Qiu*

Jiangsu Co-Innovation Center of Efficient Processing and Utilization of Forest Resources, College of Chemical Engineering, Nanjing Forestry University, Nanjing 210037, China.

*Corresponding author.

Email: jhq@njfu.edu.cn

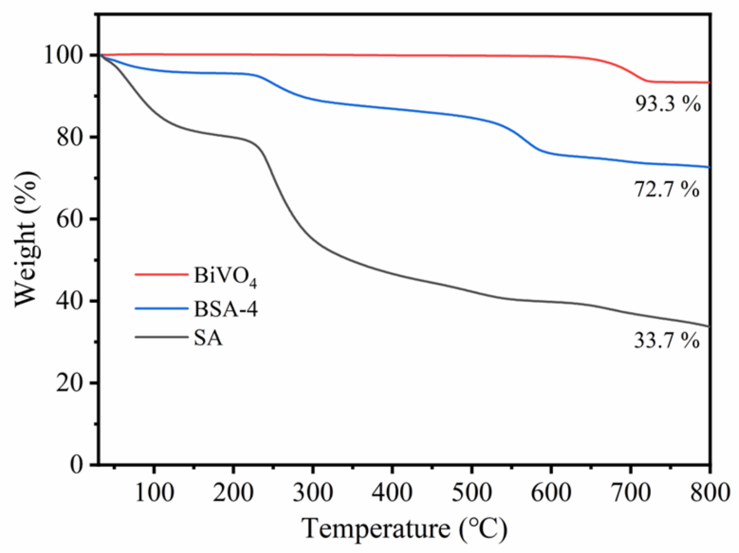

Figure S1. Thermogravimetric analysis (TGA) under a nitrogen atmosphere of different samples.

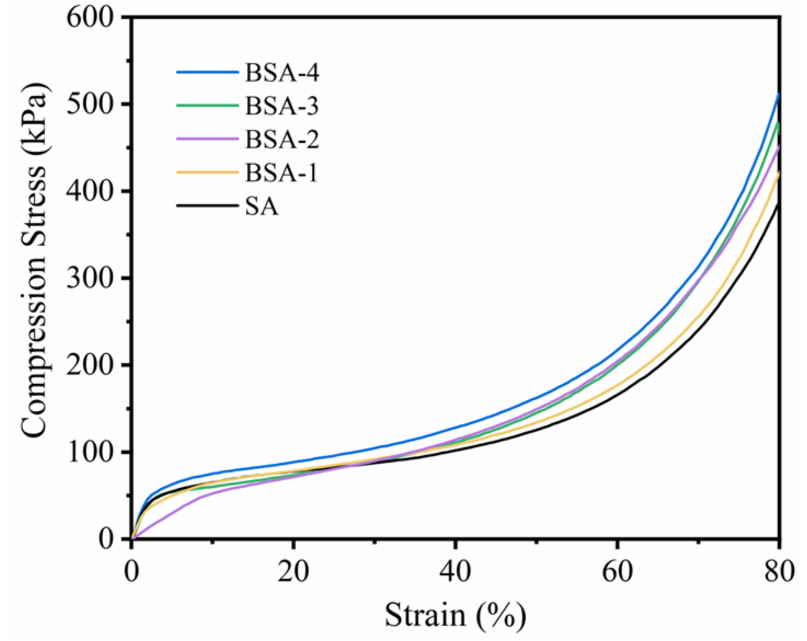

Figure S2. Stress-strain curves of different samples.

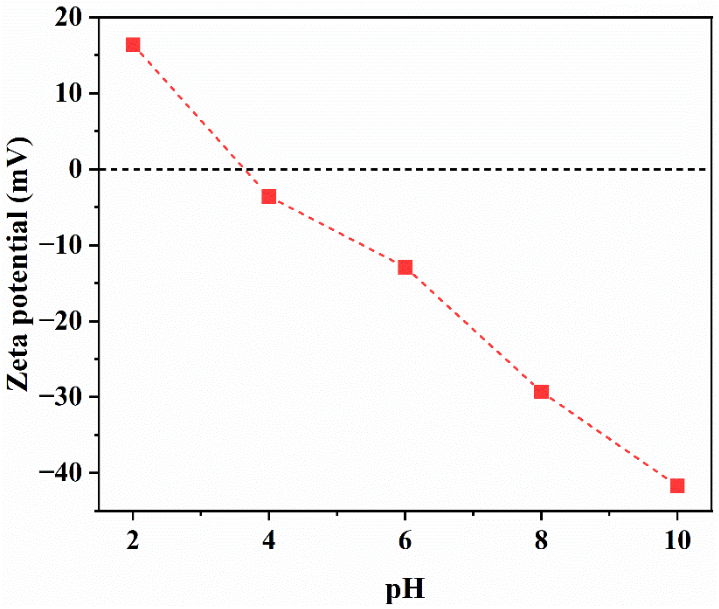

Figure S3. Zeta potentials of $BiVO_4$ under different pH values.

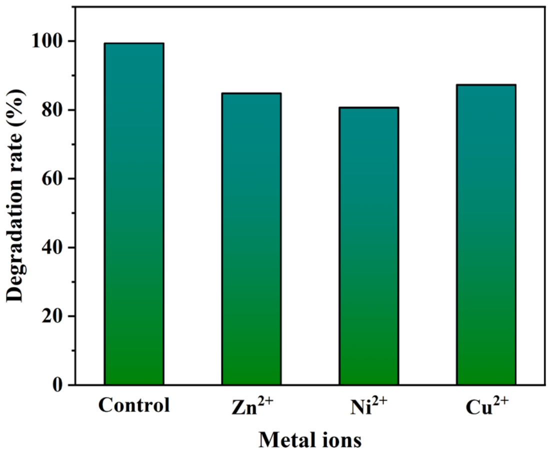

Figure S4. Photocatalytic degradation rates of BSA-4 for RhB in the presences of various heavy metal ions.
